# Defining the signature of deformable and infectious *Plasmodium falciparum* gametocytes

**DOI:** 10.12688/wellcomeopenres.25935.3

**Published:** 2026-05-14

**Authors:** Deepali Ravel, Sanna Rijpma, Dario Beraldi, Wouter Graumans, Lisette Meerstein-Kessel, Kathryn crouch, Armin Passecker, Geert-Jan van Gemert, Sara Lynn Blanken, Emmanuel Arinaitwe, Stoter Rianne, Priscilla Ngotho, Daniel Neafsey, Till Voss, Martijn Huijnen, Teun Bousema, Matthias Marti

**Affiliations:** 1 Immunology and Infectious Diseases, Harvard University T H Chan School of Public Health, Boston, Massachusetts, USA; 2 Medical Microbiology, Radboud University Medical Centre, Nijmegen, The Netherlands; 3 Infection and Immunity, University of Glasgow, Glasgow, Scotland, UK; 4 Swiss Tropical and Public Health Institute, Allschwil, Switzerland; 5 University of Basel, Basel, Switzerland; 6 Infectious Diseases Research Collaboration, Kampala, Uganda; 7 Vetsuisse and Medical Faculties, University of Zurich, Zurich, Switzerland; 8 Broad Institute of Harvard and MIT, Boston, USA; 9 Medical Biosciences, Radboud University Medical Centre, Nijmegen, The Netherlands; 10 Department of Infection Biology, London School of Hygiene & Tropical Medicine, London, UK

**Keywords:** Malaria, Plasmodium falciparum, transmission, gametocyte, infectivity, deformability

## Abstract

**Background:**

One of the major challenges for malaria elimination is combating the highly efficient spread of the disease. Despite progress in understanding the development of malaria transmission stages, there remain many unanswered questions about how gametocytes transition from being immature to infectious. In *Plasmodium falciparum,* immature gametocytes are rigid and sequester outside of circulation in the extravascular space of the bone marrow, while deformable, mature stages are found in circulation and transmitted to mosquitoes. It is currently unclear whether deformable gametocytes are immediately infectious to mosquitoes, or whether they undergo activation upon release into circulation.

**Methods:**

We used a combination of phenotypic assays and transcriptional analysis to define the transition from immature non-infectious to mature infectious gametocyte. Specifically, we associated gene expression with distinct phenotypic traits: gametocyte deformability assessed by microsphere filtration, and gametocyte infectivity assessed by exflagellation and mosquito feeding assays.

**Conclusions:**

Our data revealed major transcriptional differences between input and deformable (i.e., filtered) gametocytes, but high similarity between deformable and infectious gametocytes. In combination with exflagellation and transmission results upon mosquito feeding assays, this suggests that deformable gametocytes are immediately infectious upon release from the bone marrow. The transcriptional analysis revealed a comprehensive set of infectivity markers that can be utilized to track gametocytes during their development and serve as diagnostic tools to map the human infectious reservoir.

## Plain Language summary

Malaria spreads when mosquitoes ingest specialized malaria life stages, called gametocytes, from infected people. For the deadliest malaria parasites, *Plasmodium falciparum*, gametocytes undergo a long maturation process. Young gametocytes of this parasite are stiff and hidden in the bone marrow. Following maturation, the gametocytes become more flexible and are released into the bloodstream. It remained unclear whether these flexible gametocytes are immediately infectious to mosquitoes or need further maturation.

The current study examined changes in the gametocyte, from stiff to deformable and from non-infectious to infectious, in relation to gene activity. The results show that once gametocytes become flexible and enter the bloodstream, they are already infectious. The study also identified genetic markers linked to infectivity, which could help track malaria transmission and improve tools to identify people who can spread the disease.

## Introduction

Despite considerable success in reducing malaria burden in recent decades, there are still over 600,000 deaths due to malaria each year ^1^. Following the wide-scale deployment of insecticide treated nets and effective antimalarials, a decline in malaria burden of more than 50% was observed in countries like The Gambia, Kenya and Tanzania ( Noor *et al.*, 2014), stimulating ambitious malaria elimination efforts ( Moonen *et al.*, 2010). These trends are not universal ( Noor *et al.*, 2014), however, and malaria elimination will not be achieved in the vast majority of African settings with currently available tools ( Alonso *et al.*, 2011; Griffin *et al.*, 2010). One of the major challenges for malaria elimination is combating the highly efficient spread of the disease.

Malaria transmission from humans to mosquitoes depends on the presence of mature gametocytes in the peripheral blood. Gametocytes are sexual stage parasites that develop from their asexual progenitors at a low rate, typically comprising <1% of the total parasite population for *P. falciparum* ( Bousema & Drakeley, 2011). Once ingested by mosquitoes during a blood meal, mature male and female gametocytes activate and fuse to form zygotes that penetrate the midgut wall and develop into oocysts, giving rise to sporozoites that migrate to the mosquito salivary glands and render the mosquito infectious to humans, thus completing the parasite lifecycle.

The development of molecular gametocyte detection tools based on gametocyte-specific mRNA such as *pfs25* (PF3D7_1031000), *pfccp4* (PF3D7_0903800), *pfs230p* (PF3D7_0208900), PF3D7_0630000 and *pfmget* (PF3D7_1469900) has uncovered that gametocytes are commonly present in *P. falciparum* infections at densities below the microscopic threshold for detection ( Tadesse *et al.*, 2019). Submicroscopic gametocyte densities commonly result in mosquito infections ( Babiker *et al.*, 2008). Likelihood of mosquito infection is positively correlated with gametocyte density but plateaus at higher densities ( Bradley *et al.*, 2018). There is neither an obvious lower threshold gametocyte density below which transmission is absent nor an obvious threshold density above which infectivity is guaranteed ( Barry *et al.*, 2021; Bousema *et al.*, 2014; Churcher *et al.*, 2013). Hence, there may be additional factors apart from gametocyte density in the peripheral blood circulation that deter mosquito infectivity.

In *P. falciparum,* immature gametocytes (Stages I-IV) sequester in host tissues, primarily bone marrow, while mature stages (Stage V) are found in blood circulation ( Aguilar *et al.*, 2014; De Niz *et al.*, 2018; Joice *et al.*, 2014). During maturation, they undergo marked changes in morphology and deformability. Gametocytes are initially round forms indistinguishable from asexual stages (termed Stage I). They then progress through D-shaped transition stages (Stages II/III) to an elongated spindle form (Stage IV) and then to the final crescent form observed in circulation (Stage V) ( Hawking *et al.*, 1971; Sinden *et al.*, 1978). The mechanical properties of these distinct morphological stages have been characterized, revealing decreased gametocyte deformability during Stage I-IV and restored deformability during Stage V ( Aingaran *et al.*, 2012; Dearnley *et al.*, 2012; Tiburcio *et al.*, 2012). Computational modelling based on infected red blood cell deformability predicts that immature gametocytes cannot pass through sinusoidal slits during splenic filtration ( Aingaran *et al.*, 2012; Pivkin *et al.*, 2016), agreeing with observations of immature gametocytes being sequestered. Deformability restoration in stage V gametocytes thus allows them to exit the sequestration sites and circulate in the blood stream before being taken up in a mosquito blood meal, egressing from the host cell and successfully concluding the sexual cycle. However, it is currently unclear whether gametocytes are immediately infectious to mosquitoes upon their release into the bloodstream or whether several days of further maturation are required ( Barry *et al.*, 2021; Collins *et al.*, 2018; Lensen *et al.*, 1999); the onset of infectivity is highly relevant to determine the contribution of populations to the human infectious reservoir for malaria. In this study, we investigate the dynamics and transcriptional signatures of deformability changes in *P. falciparum* gametocytes and their relation to mosquito infectivity.

## Results

### Quantifying deformability and mosquito infectivity during gametocyte development

**Figure 1.**
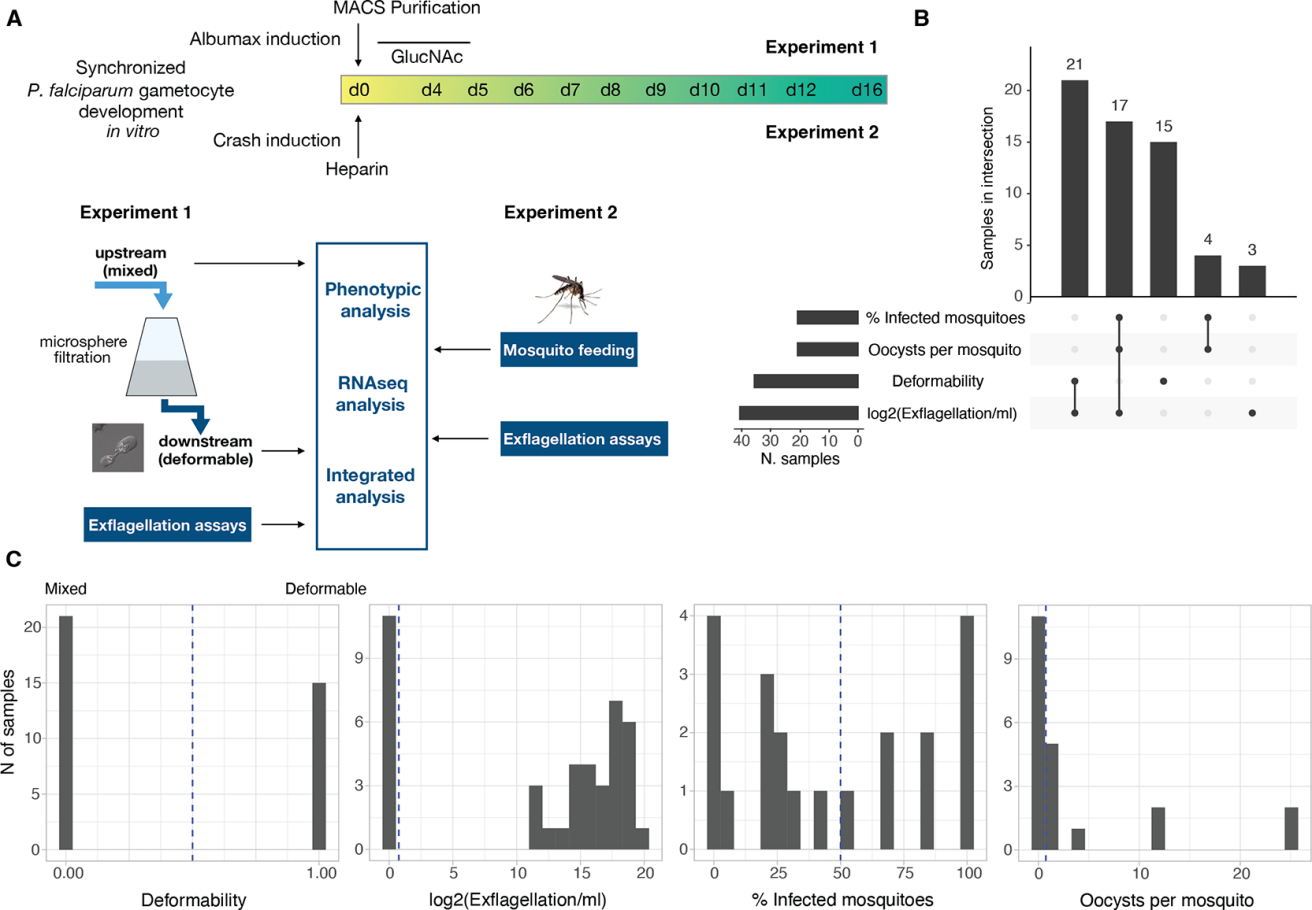
Experimental setup and data set overview. A. Schematic representation of the two separate experiments. Two independent experimental approaches focusing on either deformability (experiment 1) or mosquito infectivity (experiment 2) are used as input for integrated phenotypic and transcriptional analysis. B. Characteristics of the dataset. Number of samples in each trait (bar plot on the left) and number of samples measured for one or more traits (bar plot on top). There are 60 samples in total. C. Data distribution across traits. For deformability, 0 means mixed (upstream sample) and 1 deformable (downstream sample). Note that: The data for Infected mosquitoes and Oocysts per mosquito were collected from the same samples. A subset of samples have data for all the three infectivity traits, but no sample has data for all infectivity traits and for deformability *.*

In a first series of experiments, we aimed to elucidate the dynamics of deformability, exflagellation and mosquito infectivity during gametocyte maturation. Two separate sets of experiments were performed on gametocyte samples across stage IV to V transition and during stage V maturation (Day 6-12 of synchronised gametocyte cultures) ( Ravel, 2017). In the first set, gametocyte deformability was measured at 24-hour intervals by a microsphere filtration assay that accurately mimics splenic passage ( Lavazec *et al.*, 2013). In this assay, the input material (i.e. the unfiltered, upstream sample) includes all gametocytes, while the output (filtered, downstream sample) includes only deformable gametocytes, enabling separation of deformable gametocytes and measurement of deformability at each time point. In parallel, RNA was isolated for sequencing from input synchronized culture material and downstream filtration samples (≥ day 9 contained sufficient gametocytes). Furthermore, at each time point, male gamete exflagellation was analysed (from upstream samples only, as there was insufficient yield from the downstream samples) and in parallel RNA was isolated. Giemsa smears of parasite cultures were also made to examine the morphology of synchronized culture and downstream filtration deformable samples. A summary of the samples harvested, and the raw data collected in this time course experiment is presented in **Table S1**. In a second set of experiments, gametocyte infectivity was measured. Analysis of male exflagellation was performed and mosquito infectivity in standard membrane feeding assays (SMFA) was evaluated with the proportion of infected mosquitoes (i.e., oocyst prevalence) and oocyst numbers per mosquito (i.e., oocyst density) as SMFA endpoints. In parallel, RNA was harvested from all cultures ( **Table S1**). An overview of the experimental setup, samples and data points collected in this study is shown in Figure 1.

**Figure 2.**
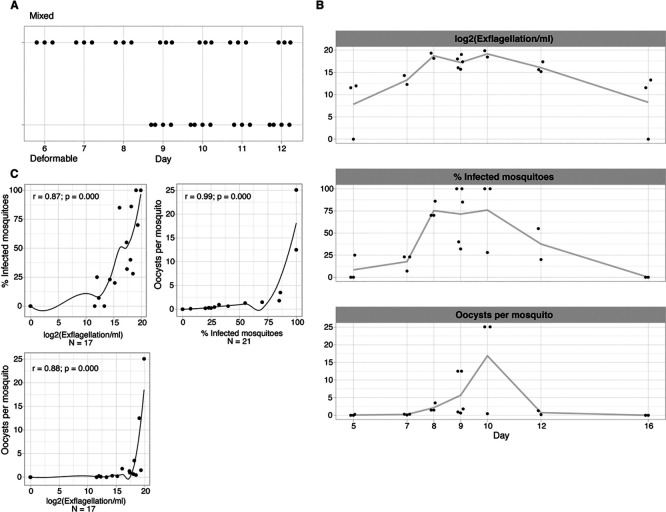
Association between deformability and infectivity traits. A. Temporal trend of exflagellation vs deformability. Mixed = upstream sample; deformable = downstream sample. x-axis is number of days since gametocyte induction. B. Temporal trend of infectivity traits. The line connects the mean of each time point. x-axis is number of days since gametocyte induction (jitter on the x-axis to prevent overplotting). C./D. Pairwise correlations between individual infectivity traits.

Analysing the four traits (i.e., gametocyte deformability, male exflagellation, oocyst density and prevalence) over time revealed a similar dynamic during gametocyte development, with peak activity for all four traits at day 8-12 of synchronised gametocyte cultures ( Figure 2A,B). To quantify the association between individual traits we used Spearman’s correlations ( Figure 2C,D). The strongest correlation was observed between the two mosquito infection traits (oocyst density *vs* prevalence, r = 0.99) that are known to be closely related ( Churcher *et al.*, 2012), followed by the correlation of either of these two traits with male gametocyte exflagellation (r = 0.82 for oocyst prevalence, r = 0.81 for oocysts density). Altogether, these data demonstrate that traits are positively correlated with each other and coincide temporally during final gametocyte maturation. However, correlations between these phenotypic assays do not reveal whether deformable gametocytes are indeed functionally equivalent to those that can exflagellate and hence are infectious gametocytes.

### Differential gene expression analysis identifies signatures of deformability and infectivity traits

**Figure 3.**
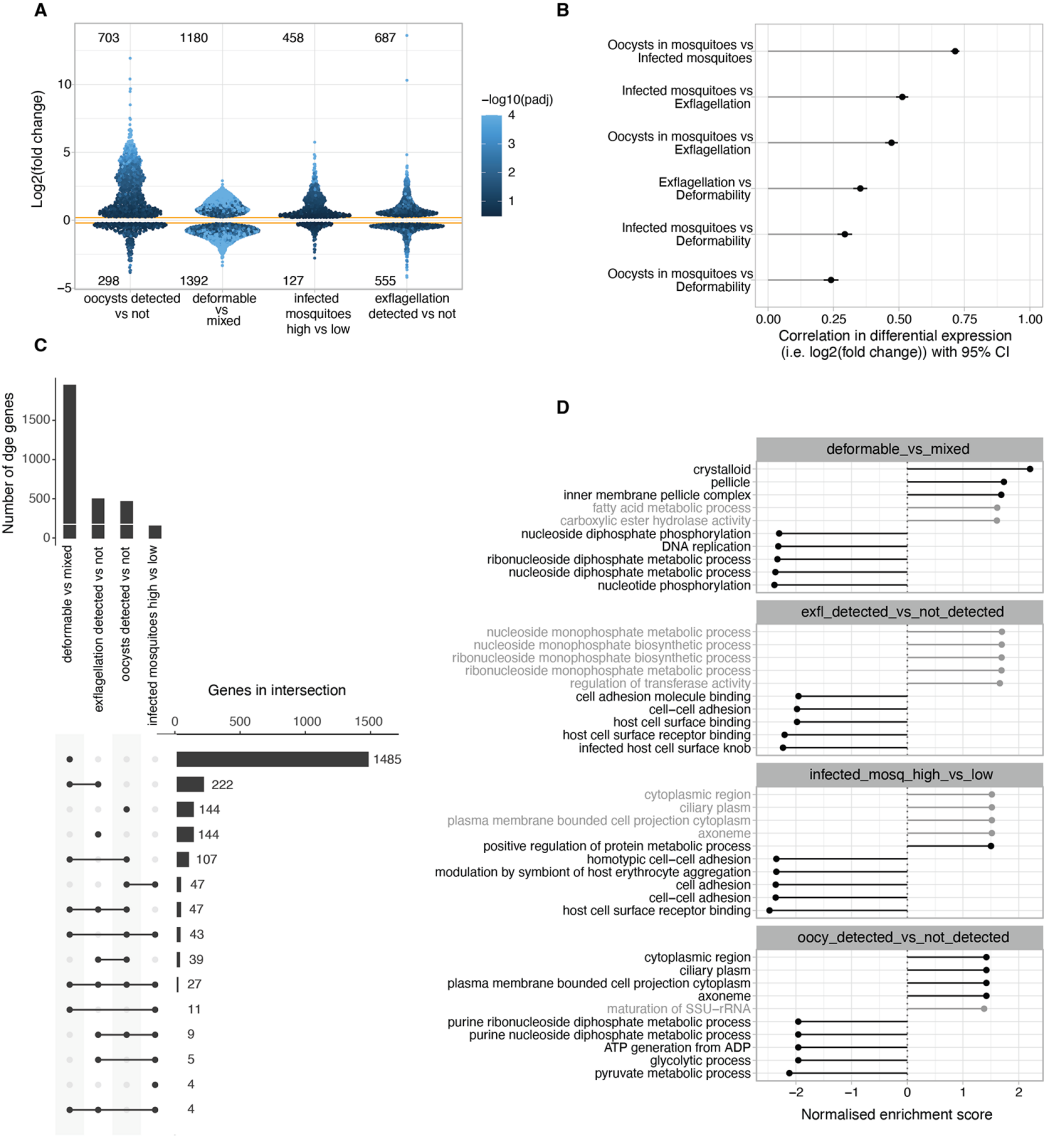
Differential gene expression across deformability and infectivity traits. A. Change in gene expression (y-axis) in relation to the dichotomised study traits. For deformability, the change is the difference between mixed (upstream) and deformable (downstream) samples. For the other traits, the phenotype value was converted to z-score to make the change in expression approximately comparable across traits. For each gene the log fold2 change is the result of one standard deviation change in trait value. Shown is DGE with p<0.05 and log FC>0.2. Each point is a gene. B. Correlation between change in gene expression across dichotomised traits from A. The correlation in expression reflects the correlation between traits. C. Overlap in DGE across traits. Inclusion requires at least 50% more expression in one condition *vs* the other and with adjusted *p*-value < 0.05 across traits. D. Gene set enrichment analysis (GSEA) of gene ontology terms. Top enriched GO terms in genes ranked by fold-change. Terms with FDR > 0.05 grayed out. A term has positive enrichment if genes in that term are associated with a positive change in change expression.

In a next series of experiments, we analysed the transcriptional profiles of all samples and used the differential gene expression (adjusted *p*-value <0.05) within and across our study traits (i.e., gametocyte deformability, male exflagellation, oocyst density and prevalence) to identify specific transcriptional markers and signatures for each of the dichotomized traits. Even if this dichotomization loses granularity, it provides changes in expression that are easier to interpret and more comparable across traits. RNAseq data were corrected for batch variation ( **Figure S1,** see also methods) and differential gene expression analysed for each trait ( Figure 3A and **Table S2**). Both deformability and exflagellation traits were associated with large numbers of differentially expressed genes ( *p*-value <0.05). Specifically, 2572 genes were differentially expressed between deformable and mixed input samples (1151 genes with log _2_ fold change ≥ ±1), and 598 genes between exflagellating and non-exflagellating samples (323 genes with log _2_ fold change ≥ ±1). In contrast, only 156 (140 genes with log _2_ fold change ≥ ±1) and 476 (431 genes with log _2_ fold change ≥ ±1) genes showed significant differences in oocyst density and prevalence traits, respectively. The reason for this difference in the number of differentially expressed hits across infection traits may be biological but it could also have technical reasons, as parasite populations lose synchronicity with increasing duration of the experiment (see also variation across biological replicates in Figure 2A,B). To quantify the overlap in differential gene expression (DGE) hits across dichotomized infection traits, we considered genes that were at least 50% more expressed in one condition *versus* the other and with an adjusted *p*-value < 0.05 across traits ( **Table S3**). A number of genes were upregulated in deformable, exflagellating and infectious gametocytes whilst downregulation was observed for a considerably smaller number of genes in association with these phenotypes. Overall, 261 genes show positive DGE correlation with deformability and either exflagellation or mosquito infectivity traits. A similar number of 278 genes only show positive DGE correlation with exflagellation and/or mosquito infectivity trait but not with deformability. In contrast, only 10 genes show positive DGE with deformability and negative correlation with either exflagellation or mosquito infection traits ( Figure 3B,C). Finally, we identified a shortlist of 92 candidate hits that were downregulated in deformable samples but showed a positive DGE association with either exflagellation or mosquito infection traits ( **Table S3**). If indeed infectious Stage V gametocytes represent a transcriptionally and physiologically different state from deformable Stage V gametocytes, then some of these candidate hits could represent this unique infectious state.

Gene set enrichment analysis (GSEA) of Gene Ontology (GO) terms revealed significant depletion of gene expression related to host cell remodelling and cell adhesion associated with exflagellation and mosquito infection traits ( Figure 3D, **Table S4**). This likely reflects the fact that gene expression prepares for gametes and mosquito stages that are extracellular and therefore do not need infected RBC surface adhesins. On the other hand, deformable gametocytes showed enrichment in gene expression related to mature gametocyte and gamete features such as the crystalloid body and apical organelles.

### Transcriptional network analysis identifies clusters associated with deformability and infectivity traits

**Figure 4.**
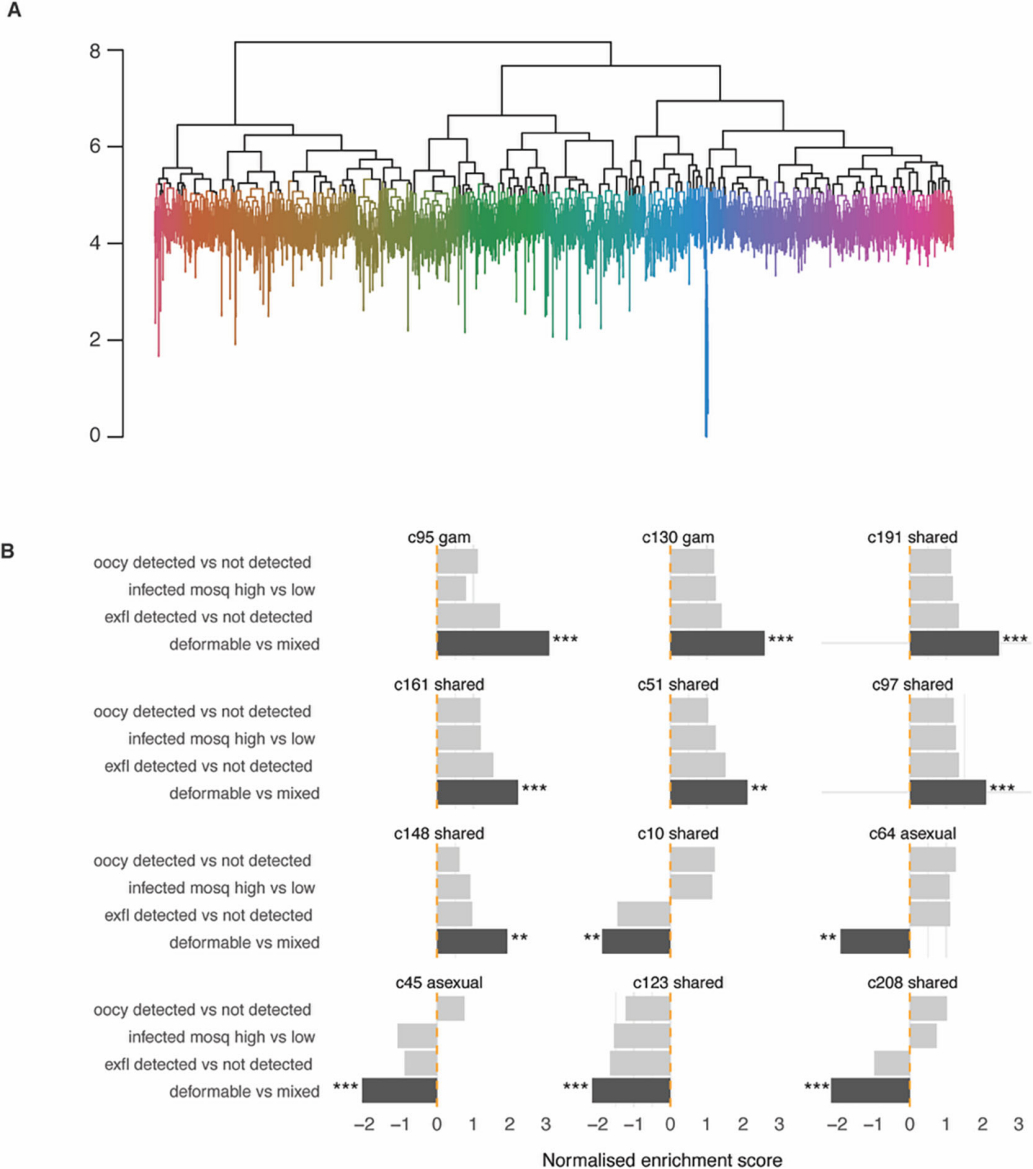
Transcriptional clusters associated with deformability and infectivity traits. A. Dendrogram of transcriptional clusters. Dendrogram clustering genes according to their expression in sample from time-course experiments and *in vivo* datasets. To obtain discrete clusters for further analyses, the dendrogram was cut at the 95 ^th^ quantile of the gene heights. Colors highlight these clusters. B. Gene clusters associated with gene expression changes. Associations are determined by gene set enrichment analysis. Clusters from time course analysis enriched in genes associated with infectivity and deformability. The clusters plotted here have an adjusted *p*-value < 0.01 for enrichment in at least one trait. The reported effect size is positive or negative depending on whether the genes in a cluster tend have positive or negative fold-change associated to the study trait. The stars and colours indicate the level of significance (*** *p*-adjusted < 0.001, ** < 0.01).

We have previously generated a transcription network in *P. falciparum* to define clusters of co-expressed genes during asexual and gametocyte development and their enrichment in specific pathways ( Pelle *et al.*, 2015). Here we aimed to generate such a network to identify clusters (and therefore gene sets) that are enriched in the four characteristics associated with parasite infectivity. For this purpose, we used the publicly available microarray expression data ( Hu *et al.*, 2010; Le Roch *et al.*, 2003; Llinas *et al.*, 2006; Young *et al.*, 2005) and added a published gametocyte time course data set ( Zhu *et al.*, 2018). The resulting network contains 235 transcription clusters with a size of 5 (set as minimum gene number) to 87 genes ( Figure 4A and **Table S5**). The transcription network consists of gametocyte-specific clusters, asexual clusters and those shared between gametocytes and asexual stages, as defined based on stage-specific transcript and protein expression pattern in published data sets (PlasmoDB release 68). Our findings highly overlap with the networks we previously published ( Pelle *et al.*, 2015) (see **Table S5**). A subset of clusters was significantly enriched in genes associated with DGE in at least one of our four traits. Specifically, two gametocyte-specific clusters (95 and 130) and six clusters shared with the asexual stage (51, 97, 148, 161, 162, 191) contained genes whose expression was positively associated with deformability and exflagellation ( Figure 4B). Together, the two gametocyte-specific clusters contain many of the known mature gametocyte marker transcripts, including *pfs25*, *pfs28, Pfs230p, pfccp4*, *pfmget* and *pfg377* ( **Table S2)**. Indeed, the mean transcript peak of these two clusters is in mature gametocytes in the published gametocyte time course ( **Figure S2**).

### Analysis of putative infectivity markers in cohorts of naturally infected individuals

**Figure 5.**
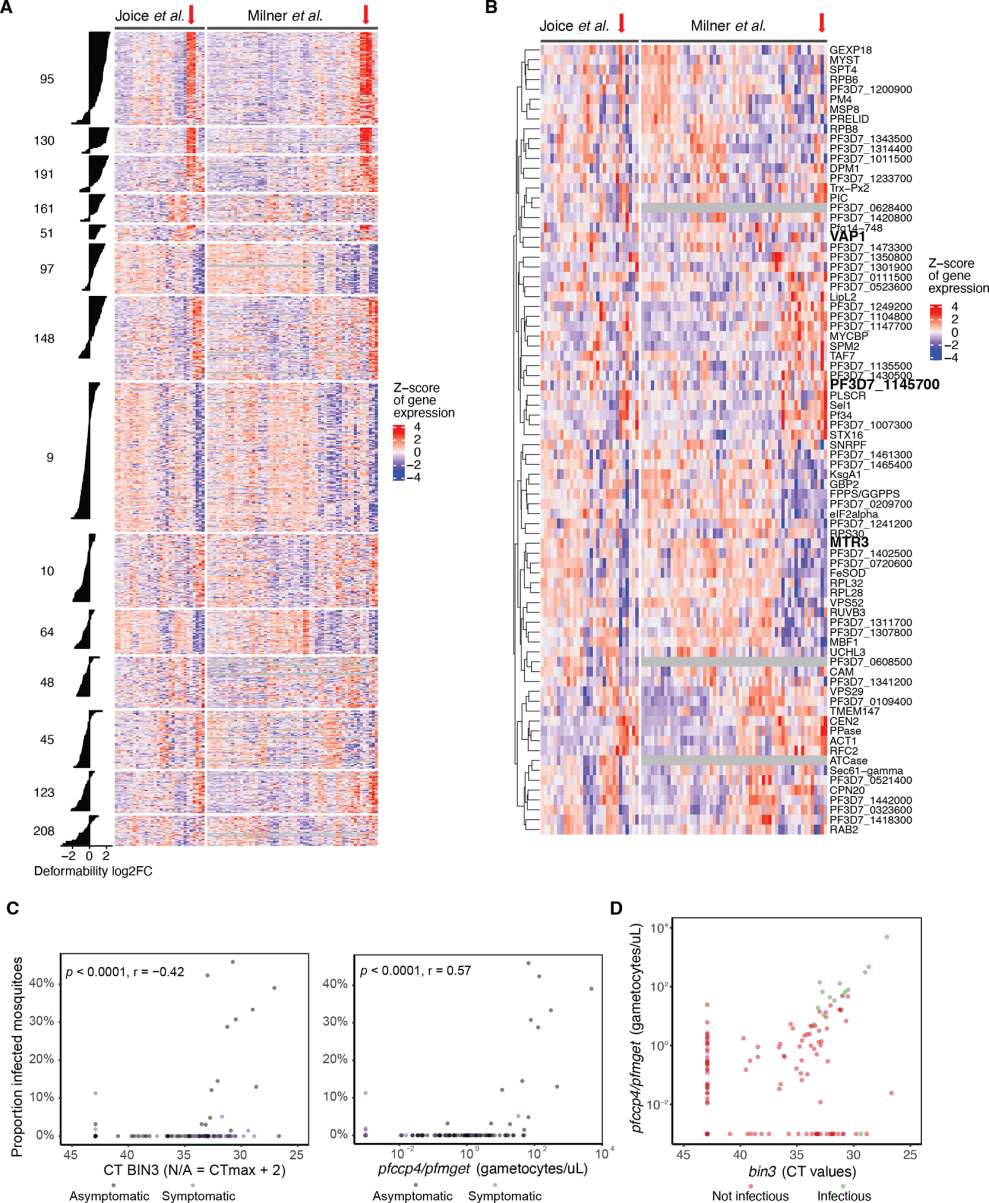
Detection of putative infectivity markers in patient samples. A. Heatmap of gene expression across patient cohorts with select clusters (DGE in deformability) with associated bar plots for DGE. Clusters 95, 130 and 191 are detectable in the same subset of patients. Others (161, 97, 148) less so, even though they show a similar but less strong DGE trend for deformability. B. Heatmap of candidate gene expression across patient cohorts. Select 92 candidate genes with negative DGE correlation between deformability versus exflagellation and/or mosquito infection traits. Black and red frames indicate candidates that were further analysed by qRT-PCR. C. Validation of *bin3* in patient samples. Left panel: Scatter plot of *bin3 (*PF3D7_1145700 *)* CT values versus the proportion of infected mosquitoes. Right panel: Scatter plot of gametocyte density based on combined female ( *pfccp4*) and male ( *pfmget*) marker CT values versus the proportion of infected mosquitoes. D. Correlation between transcript abundance of the novel bin3 gametocyte marker and established gametocyte assays. Samples are from a cohort of Ugandans naturally infected with *P. falciparum* (as figure 5C). CT values for *bin3* are presented on the X-axis; gametocyte density based on the combined *pfccp4/pfmget* multiplex qPCR is presented on the Y-axis. Colors indicate whether the blood sample resulted in mosquito infection (green) or not (red). Of note, one sample that was infectious to mosquitoes but had no measurable *pfccp4/pfmget* transcripts showed a low CT-value for *bin3*, indicating relatively high gametocyte levels for this marker. Red arrow in A and B shows patient samples with high cluster 95, 130, 191 gene expression (A) and candidate gene expression (B).

To determine whether putative infectivity markers identified in this study are expressed during human infection, we analysed parasite gene expression data from malaria patient blood. For this purpose, we selected published gene expression data sets from one cohort of uncomplicated malaria in Senegal and one cohort of cerebral malaria in Malawi ( Pelle *et al.*, 2015). The majority of genes from both gametocyte-specific clusters 95 and 130, and a subset of genes from the shared cluster 191 (asexual and gametocyte expression), showed high expression in the same subset of patient samples from both cohorts ( Figure 5A, **Table S5**). Metadata from these two cohorts reveal that these patient samples are amongst a subset of samples that have detectable gametocyte levels by blood smear, as previously observed ( Pelle *et al.*, 2015), indicating the limited sensitivity of these early gene expression studies ( Joice *et al.*, 2013; Milner *et al.*, 2012). Genes from all other gametocyte clusters were only minimally expressed or undetectable in these blood samples, in agreement with their peak expression in immature gametocytes ( **Figure S2**). Of note, these cohorts of patients from Malawi and Senegal had data on gametocyte carriage but not on infectivity to mosquitoes.

Importantly, a subset of the 92 candidate genes that were downregulated in deformable samples but showed a positive DGE association with either exflagellation or mosquito infection traits also showed high expression in the same subset of patient samples as genes from clusters 95 and 130 ( Figure 5B). To independently validate these data, we selected three representative candidates of the 92 individual genes ( **Table S4**) for qRT-PCR analysis ( Figure 5C). Specifically, we selected i) PF3D7_0209200 (annotated as putative exosome complex component MTR3), with a negative DGE for deformability and positive DGE for oocyst detection in mosquitoes; ii) PF3D7_0936500 (annotated as virulence associated protein, VAP1), with a negative DGE for deformability and a positive DGE for exflagellation and oocyst detection in mosquitoes; and iii) PF3D7_1145700 (annotated as putative bicoid-interacting protein BIN3), with a negative DGE for deformability and a positive DGE for oocyst detection and infection prevalence in mosquitoes. Whilst *mtr3* and *vap1* showed similar expression in ring and Stage V gametocyte stages in a pilot experiment, *bin3* was highly expressed in Stage V while only detectable at high level of input material in ring stages ( **Table S6**). We thus prioritized *bin3* for optimization and validated this target on blood samples from an asymptomatic malaria-infected cohort in Uganda with known gametocyte density data and whose venous blood was offered to mosquitoes in direct membrane feeding assays, thus providing a direct measure of mosquito infectivity data ( Rek *et al.*, 2022). *Bin3* levels were highly correlated with male and female gametocyte levels, as assessed by *pfccp4* and *pfmget* qRT-PCR, and mosquito infection rates by direct membrane feeding assays ( Figure 5C, D). We found no indications that *bin3* levels were more closely associated with mosquito infection prevalence compared to *pfccp4* and *pfmget.* Higher CT values reflect lower transcript numbers; the correlation is thus in the same direction for bin3 as that for *pfccp4/pfmget*. We also observed a strong correlation between the two measures of gametocytes ( Figure 5D).

## Discussion

Understanding gametocyte commitment, maturation and infectivity is essential to successfully undertake the major challenge in malaria elimination efforts, i.e. identifying infectious individuals and preventing onward malaria transmission. For *P. falciparum,* human infectivity to mosquitoes follows a long gametocyte maturation process during which gametocytes are sequestered with uncertainties about the infectiousness of stage V gametocytes immediately following their release into the bloodstream. In this study we ask whether there is a specific transcriptional profile that sets aside infectious gametocytes from deformable gametocytes.

A previous study has described the dynamics of mosquito infectivity during *in vitro* gametocyte development, revealing a peak around days 10-12 of gametocyte maturation (coinciding with the maximum proportion of mature Stage V gametocytes) before infectivity gradually drops ( Lensen *et al.*, 1999). More recent *in vivo* evidence suggested that mosquito infectivity is lower early in infection, possibly due to higher levels of inflammation or lower infectivity of gametocytes immediately upon release into circulation ( Barry *et al.*, 2021). Here we aimed to complement these phenotypic data with transcriptional profiling and define the transcriptional signatures that drive or delineate deformability and mosquito infectivity changes in gametocytes.

To this purpose, we optimized a biomechanical assay by which deformable and stiff gametocytes could be separated and used this assay to measure gametocyte deformability and exflagellation assays that confirm transmission potential of male gametocytes. In parallel, we performed a gametocyte time course to measure exflagellation and gametocyte infectivity using mosquito feeding assays. For each sample from these two experimental setups, we collected an aliquot for RNAseq to correlate phenotypic traits with parasite transcriptional signatures. In agreement with the previous study ( Lensen *et al.*, 1999) we observe a peak in infectivity traits at around 10 days of gametocyte maturation, before infectivity declines. Importantly, both deformability and exflagellation traits also peak around day 10 and exflagellation rates decline thereafter (while deformability does not), demonstrating that the dynamics of all four traits coincide. Indeed, we observed strong positive correlations for both mosquito infectivity traits (oocyst prevalence and density) and exflagellation (>0.75). Transcriptional profiling of the samples used for phenotypic analysis confirmed these positive correlations. To investigate patterns of gene expression across traits we performed GSEA of GO terms, revealing some interesting differences. Notably, female gamete traits such as the crystalloid body were enriched in deformable gametocytes, while male traits such as DNA replication (occurring during gamete exflagellation) and cilium genes were depleted in these gametocytes that were not yet exposed to triggers for gamete formation. This likely reflects the faster maturation time of female gametocytes compared to males. Indeed, known female gametocyte marker gene expression increases faster than male marker gene expression in the deformability data set and in the *in vitro* time course, and the difference of mean female *vs* male marker gene expression across multiple markers stabilises only during final gametocyte maturation ( **Figure S3**).

Transcriptional network analyses identified three clusters enriched in deformability, exflagellation and mosquito infectivity traits. These three clusters contain many of the known mature gametocyte markers, including those used for molecular diagnostics of gametocyte load in patients such as *pfs25*, *Pfs230p*, *pfccp4* and *pfg377* ( Tadesse *et al.*, 2019). Indeed, genes of these clusters (but not of other clusters) are also detectable across blood samples from two cohorts of malaria patients ( Pelle *et al.*, 2015). Importantly we also identified a smaller set of genes with significantly higher expression in exflagellating and/or infectious gametocytes compared to immature (non-deformable) gametocytes and validated one of these genes by qRT-PCR in blood samples from malaria patients. This novel molecular diagnostics marker, *bin3,* was positively associated with the proportion of mosquitoes that became infected when feeding on this blood sample. We found, however, no indications that infectivity was better predicted by *bin3* transcript numbers than by established markers of male and female gametocyte abundance *.* This supports previous observations that mature circulating gametocyte biomass, as quantified by established male and female gametocyte markers, is a fair predictor of infectivity ( Bradley *et al.*, 2018). Moreover, the current study did not include an extensive optimization of qPCR for other gametocyte markers. It is therefore possible that other markers, as identified in this study, are equally good or superior predictors of infectivity.

Altogether our data suggest that deformable gametocytes are immediately present in circulation and likely infectious, as they share the same phenotypic and transcriptional profile as exflagellating and infectious gametocytes. Our markers did not explain observations that we and others made that there is variation in infectivity between batches of gametocytes that morphologically appear mature with an apparent peak in infectivity around day 10-12 following induction.

There are some limitations to our study. First, our experiments and those previously performed ( Lensen *et al.*, 1999) were done under controlled *in vitro* conditions. It remains to be determined whether the observed rapid decline in mature gametocyte infectivity is physiological or the result of *in vitro* culture condition and/or parasite culture adaptation. In natural infections where asexual parasitaemia is interrupted by treatment with gametocyte-permissive antimalarial drugs, gametocytes remain detectable for over 28 days and infectious for at least 21 days ( Collins *et al.*, 2018; Mahamar *et al.*, 2024). With declining gametocyte densities over time – and a logically associated decline in infectivity – it is complicated to unequivocally demonstrate variations in per gametocyte infectivity over the course of gametocyte carriage. Notably, a recent single cell RNA sequencing study of circulating gametocytes in patient samples identified two subpopulations of mature gametocyte stages based on RNA content (independent of sex), however the biological relevance of this finding remains to be determined ( Dogga *et al.*, 2024). Second, we have correlated phenotypes only with transcriptional signatures while not using other parameters that are highly stage-specific (e.g., proteomics, metabolomics, epigenetics, post-translational modifications). It is therefore possible that such parameters could reveal distinct profiles between deformable and infectious gametocytes. However, the strong positive correlation between phenotypic traits and transcriptional profiles strongly suggests that deformable gametocytes are immediately infectious and hence transmission competent. Lastly, our deformability assays and infectivity assays were done as separate sets of experiments in independent laboratories. This reflects the specialised nature of these experiments, making it technically challenging to analyse one comprehensive set of culture samples where deformability and (all) infectivity assays were performed consecutively.

In conclusion, we present transcriptional profiles that differentiate gametocytes with low and high deformability phenotypes. Our data suggest that deformable gametocytes are transcriptionally similar to infectious gametocytes. We observed no compelling set of genes that identifies infectious gametocytes beyond (partially known) markers of gametocyte maturity. One newly identified marker sensitively detects gametocytes in naturally infected individuals and predicts transmission potential with similar performance compared to established markers of the biomass of mature gametocytes.

## Materials and methods

### Ethics statement

RNA samples were used from a previously published cohort study ( Rek *et al.*, 2022). Written informed consent was received from all individuals participating in that study; parents or guardians provided informed consent for children, with children aged 8 years and above providing additional assent. The Uganda National Council for Science and Technology, Makerere University School of Medicine (REC REF 2019-134), the University of California San Francisco (#19-28606), and the London School of Hygiene and Tropical Medicine provided ethical approval for the study. Experiments with *in vitro* cultured parasites and *Anopheles* mosquitoes at Radboud University Medical Center were conducted following approval from the Radboud University Experimental Animal Ethical Committee (RUDEC 2009-019, RUDEC 2009-225).

***Plasmodium falciparum in vitro* culture**

*Plasmodium falciparum* parasites were cultured as published previously with slight modifications (Trager and Jensen, 1976) ( Ravel, 2017). Briefly, cultures were grown at 4% hematocrit in O+ human red blood cells (Research Blood Components, Boston, MA, USA; Interstate Blood 61 Bank, Memphis, TN, USA); culture media consisted of RPMI 1640 supplemented with 25 mM HEPES (EMD Biosciences), sodium bicarbonate (Sigma), gentamycin, 50mg/l hypoxanthine, and either 10% O+ human serum or 0.5% Albumax (Thermo Fisher Scientific, 11020021). O+ human serum was obtained from Caucasian male donors (Interstate Blood Bank, Memphis, TN, USA). *Plasmodium falciparum* strain NF54 ( Ponnudurai *et al.*, 1981) cultures were kept at 37°C in a modular incubation chamber (Billups-Rothenberg, MIC-101) gassed with 5% CO2, 1% O2 and balanced nitrogen (Med-Tech Gases; Airgas).

***Plasmodium falciparum* gametocyte culture and exflagellation assays**

All parasites used for gametocyte production were cultured in media containing 10% human serum.

For quantifying deformability and transcriptional profiling, gametocytes were induced as previously described ( Brancucci *et al.*, 2015), with slight modifications ( Ravel, 2017). Parasites were sorbitol synchronized twice 18 hours apart to obtain synchronous cultures. At 24 hours post invasion, media containing serum was replaced with media containing 0.5 % Albumax to induce formation of gametocytes. Media containing serum was added back at 44 hours post invasion, and parasites were allowed to reinvade under shaking conditions (55 rpm). After reinvasion, magnet purification was performed to collect ring stage parasites in the flow through and remove late asexual stages (or any gametocyte stages already present in the culture) that bound to the magnet; this culture is referred to as “Day 0”. Serum media was changed daily from Day 0 onwards and was supplemented with 50 mM GlucNAc from Day 0-5 to prevent asexual growth. To minimize perturbation and prevent spontaneous exflagellation of mature gametocytes, media changes were performed on a 37°C slide warmer beginning on Day 0.

For assessing mosquito infectiousness and transcriptional profiling, asynchronized cultures of *Plasmodium falciparum* parasites were seeded at ‘Day 0’ (10 ml, 0.75% parasitemia in 5% hematocrit). On ‘Day 3’ cultures (parasitemia between 15-22%) were spun down in 15 ml tubes and the supernatant was removed until 5 ml was left. The pellet was suspended in the medium and layered on top of a Percoll density gradient (63% in 1x PBS). Tubes were centrifuged for 20 minutes at 2000 rpm; 3 minutes acceleration and 3 minutes break. The schizont-containing interphase was carefully collected using a 3 ml syringe with blunt end needle and combined cultures reintroduced in a clean culture flask at 5% haematocrit. Medium was supplemented with 20 U/mL heparin (final concentration) 5-8 hours after synchronization. Synchronous gametocytes were harvested over the next days, with fully mature Stage V gametocytes on ‘Day 11’.

To measure exflagellation, 40 μl of a 2% haematocrit gametocyte culture was centrifuged for 25 seconds. After removal of the supernatant the pellet was resuspended in 40 μl of Fetal Bovine Serum (FBS, Invitrogen, 10270106). This sample was then incubated at 23°C in a heat block for 15 minutes. 10 μl of the sample was transferred to a hemacytometer and the number of exflagellation centers per 3x16 fields was assessed in triplicate by microscopy at 400x magnification.

### Microsphere filtration

Microsphere filtration was performed as published ( Deplaine *et al.*, 2011; Tiburcio *et al.*, 2012), with some modifications ( Ravel, 2017). To fabricate the filter, 400 mg of “Type 5” beads and 400 mg of “Type 6” beads (Heraeus Electronics, #81121077 and 81124787) were weighed and combined in an Eppendorf tube (Gesick MPM – Sn 96.5%, Ag 3%, Cu 0.5%; Type 5: 15-25 um; Type 6: 5-15 um). 800 μl of pre-warmed, complete RPMI was then added to the Eppendorf containing the beads; beads were always resuspended in the same media as cells (complete RPMI supplemented with Albumax or human serum). Beads and media were then quickly resuspended in the shortened filter tip, and the filter tip was inverted to allow the beads to settle on top of the filter. Beads were allowed to settle for at least two minutes, and the tip was then filled with complete media using a needle and syringe.

Parasite culture was diluted to ≤2% hematocrit and ≤5% parasitemia in pre-warmed, complete media. When the culture contained significant amounts of debris, cells were gently washed prior to dilution. An aliquot of this culture was collected pre-filtration to serve as the “upstream” sample (for smear or flow cytometry). For asexual stage assays, all steps were performed with media pre-warmed to 37°C in a lab space at room temperature. For gametocyte assays, all steps were performed with media pre-warmed to 37°C in a warm room maintained at 37°C. For filtration, the filter tip and a 10 ml syringe were connected to a 3 way stop cock, and the 10 ml syringe was filled with 9 ml of pre-warmed, complete media (again, the same media as cells and beads were resuspended in). With the port to the 10 ml syringe closed, a 1 ml syringe 66 was used to inject 1 ml of culture into the filter tip through the stop cock at a rate of ~100 ul/second. A syringe pump was then used to wash the filter through the stop cock with 7 ml of complete media at 1 ml/minute, and the flow through was collected as the “downstream” sample. To collect parasites retained in the filter, the beads and parasites in the filter tip were resuspended and ejected into an Eppendorf tube. The beads were allowed to settle for 1-2 minutes, and the supernatant (still containing parasites) was collected. This process was repeated 2-4 times depending on desired purity.

Upstream and downstream samples were washed in PBS and fixed for 40 minutes at room temperature in PBS containing 4% Paraformaldehyde and 0.01% Glutaraldehyde. Cells were again washed in PBS and were then stored at 4°C until processing. To measure parasitemia of these samples by flow cytometry, fixed cells were washed once in PBS and incubated with PBS containing 1:5000 SYBR Green DNA dye (Applied Biosystems, #4385612) for twenty minutes in the dark. Samples were then run on a MACSQuant VYB flow cytometer, with at least 200,000 events counted per samples. Parasitemia of upstream and downstream samples was calculated, and the retention rate was calculated as 100 * (1- (downstream parasitemia/upstream parasitemia)).

To minimize perturbation of cultures, gametocyte-containing cultures were not washed prior to filtration, and all steps were performed with media pre-warmed to 37°C in a room maintained at 37°C. After filtration, upstream and downstream parasites were collected and pelleted in a pre-warmed centrifuge (37°C) for five minutes at 500 *x g.* Giemsa smears were made for each sample. For the samples used for transcriptional profiling, supernatant was removed and parasites were resuspended in 1 ml of Buffer RLT (Qiagen, 79216) supplemented with 1% b-mercaptoethanol. Samples were then quickly frozen at -80C for RNAseq.

### Mosquito infection experiments

Feeding assays were conducted with *Anopheles stephensi* mosquitoes of the Sind-Kasur strain ( Feldmann & Ponnudurai, 1989). Mosquitoes were reared at 30°C and ~80% relative humidity with reverse day and night cycle, performed as described previously ( Ponnudurai *et al.*, 1989), with slight modifications. The blood meal was prepared in a heating block set at 37°C. Briefly, 600 μl of culture material was added to 180 μl of erythrocytes and spun down for 20 seconds at 10000 rpm using a benchtop centrifuge. The supernatant was removed, and the pellet was suspended in 150 μl of serum. The blood meal was fed using glass mini feeders to cups that contained 50 female mosquitoes. After feeding, partially fed and unfed mosquitoes were removed. Transmission was assessed microscopically by detecting oocysts on mercurochrome (1%) stained mosquito midguts 7-10 days after feeding.

### Sampling for RNA sequencing

*Plasmodium falciparum* synchronized gametocyte cultures (10 ml) were harvested per 24 hours and transferred to an incubator set at 37°C. For leucocyte depletion a Plasmodipur (Europroxima, 8011) was used to filter the material into a 15 ml tube. To retain all material the filter was rinsed with prewarmed PBS (1x). The tube with the flowthrough was spun down in a pre-warmed centrifuge (37°C) for 5 minutes at 2000 rpm. The supernatant was removed, and the pellet was dissolved in 6 mL RLT buffer with 1% 2-Mercaptoethanol. Samples were stored immediately at -80°C.

### RNA preparation

RNA extractions were performed as published ( Hoeijmakers *et al.*, 2013) *.* Samples preserved in Buffer RLT were extracted using the RNeasy Mini kit (Qiagen, 74104). They were then incubated for 30 minutes at 37°C with TURBO DNase (Thermo Fisher Scientific, AM2238) and purified/concentrated using a RNeasy Micro kit (Qiagen, 74004). RNA concentration and quality was Qubit RNA HS assay (Thermo Fisher Scientific, Q33221) and confirmed at the Harvard Medical School Biopolymers Facility (Boston, MA) using the RNA 6000 Pico kit on a BioAnalyzer 2100 (Agilent).

### RNA sequencing

RNA was extracted for each time point of the deformability and infectivity assays and submitted for SmartSeq2 (Illumina) sequencing at the Broad Institute. RNA samples were diluted to 2 μg/μl in nuclease-free double distilled H _2_O (Ambion, AM9916). Ten μl of each sample was placed in a 96 well plate to obtain a final concentration of 20 ng RNA/well. The SmartSeq2 libraries were prepared according to the SmartSeq2 protocol ( Picelli *et al.*, 2013, 2014), with some modifications. Briefly, total RNA was purified using RNA-SPRI beads. Poly(A)+ mRNA was converted to cDNA which was then amplified. cDNA was subject to transposon-based fragmentation that used dual-indexing to barcode each fragment of each converted transcript with a combination of barcodes specific to each sample. In the case of single cell sequencing, each cell was given its own combination of barcodes. Barcoded cDNA fragments were then pooled prior to sequencing.

Upon sequencing, initial analysis of the results showed sufficient sequencing quality as approximately 85% of the reads aligned to the reference genome, with more than 1,000,000 unique reads identified for each sample. Sequencing was performed using 37 base pair read lengths. With the 37 base pair reads, 84.9% of forward reads and 87.7% of reverse reads were mapped to the reference genome, and 48% of all reads were mapped uniquely. Mapping of the reads to the 3D7 reference genome was performed with the Tuxedo Tools suite (bowtie + tophat) and read counts per gene were calculated with HTseqcount. If mapping was not unique, best scoring alignment was kept. Alignment score was determined by base quality of the reads, number and positions of mismatches in the alignment, and whether forward and reverse reads could be mapped concordantly.

### Phenotype and gene expression analysis

The infection traits are continuous measurements; however, they were recoded as binary variables for the differential analysis. Exflagellation and oocysts density traits were re-coded as “non-detected” if their value was zero and “detected” otherwise. Oocyst prevalence was re-coded as “low infection” if < 50% and “high infection” otherwise.

Since the technical effect of “plate” had a noticeable influence on gene expression, we corrected the row counts to remove this effect using the function ComBat_seq in the Bioconductor package sva (R package version 3.56.0). We fitted a single DESeq model which included all the available libraries. We then used the appropriate contrast matrix to compare samples grouped according to their phenotype. Log2 fold changes were shrunk using the “ashr” method ( Stephens, 2017). The association between gene expression and study traits was performed with the DESeq2 Bioconductor package ( Love *et al.*, 2014). For identification of associations between gene ontology terms and differential expression in each of the four study traits, we applied the Gene Set Enrichment Analysis (GSEA) method implemented in the Bioconductor package clusterProfiler ( Yu *et al.*, 2012). Genes were ranked according to fold change and tested against the ontology terms available in PlasmoDB release 68. The annotation package for *P. falciparum* suitable for clusterProfiler was built using the script at https://github.com/glaParaBio/utils/tree/master/makeBioconductorAnnotationDbi.

The code repository https://github.com/glaParaBio/deformable-infectious-pfalciparum-gams contains the program versions (see requirements.txt), the in-house scripts, and the analysis workflow, the latter implemented in snakemake ( Molder *et al.*, 2021).

*Gene network generation and analysis*

Genes were clustered according to their expression profiles largely following our previously published method ( Pelle *et al.*, 2015). We clustered time-course and *in vivo* datasets from microarray experiments. The time-course datasets are from ( Hu *et al.*, 2010; Le Roch *et al.*, 2003; Llinas *et al.*, 2006; Young *et al.*, 2005; Zhu *et al.*, 2018). The *in vivo* datasets are from ( Daily *et al.*, 2007; Joice *et al.*, 2013; Milner *et al.*, 2012).

Missing gene expression values within each dataset were imputed with the k-nearest neighbour algorithm using k=10. The similarity matrix of gene expression within each dataset was obtained by first calculating all the Pearson correlation coefficients for each pair of genes and then by converting the correlation coefficient via Fisher's Z-transformation. The pairwise similarities were then averaged within the seven time-course datasets and within the three *in vivo* datasets and the two resulting matrices were in turn averaged to obtain a single pairwise gene similarity matrix. Missing values in this matrix were imputed with a strategy analogous to the k-nearest neighbour. Next, the matrix was converted to a distance matrix by computing ‘1 - x’ for each entry x. Genes were clustered by hierarchical clustering using the function hclust from R v4.3.3 with default arguments. To obtain discrete clusters, the dendrogram was cut at the 95th quantile of the tree height. The analysis code is available in scripts microarray_filtering.R and makeClusters. R in https://github.com/glaParaBio/deformable-infectious-pfalciparum-gams

### Validating novel gametocyte markers

Newly identified gametocyte markers were initially tested *in vitro* using highly synchronous parasite material, harvesting 10- and 20-hour ring-stage parasites and mature stage V gametocytes ( Tadesse *et al.*, 2017). In case there was a large difference in transcript levels between ring-stage parasites and gametocytes, at least 3 CT difference, the marker was tested on field samples. For this, a cohort of Ugandans naturally exposed to malaria was selected ( Rek *et al.*, 2022). Briefly, 182 samples from 132 Ugandans (aged 0.5-56 years) were analysed, of which 63 samples were from symptomatic and 119 from asymptomatic infections. From these samples, 11.5% (21/182) were infectious to mosquitoes.

## Data Availability

•
*Source code available from*: https://github.com/glaParaBio/deformable-infectious-pfalciparum-gams/tree/main/ ( Beraldi & Marti, 2026). •
*Archived source code at time of publication*: https://doi.org/10.5281/zenodo.18607727 ( Dariober, 2026) •
*License:* GNU Affero General Public License v3.0 All data are available on GitHub in the repository named Parasite Bioinformatics at the University of Glasgow, under project title “Deformable -infectious- pfalciparum-gams”. This project contains the following underlying data:
- gene expression data are available at https://github.com/glaParaBio/deformable-infectious-pfalciparum-gams/tree/main/ref_data/counts; - pre-filtered expression matrices and auxiliary files are available at https://github.com/glaParaBio/deformable-infectious-pfalciparum-gams/tree/main/ref_data/microarrays/rawInput. - from each dataset we removed all paralogs of the *var*, *rifin* and *stevor* variant antigen families, as listed under https://github.com/glaParaBio/deformable-infectious-pfalciparum-gams/tree/main/ref_data/microarrays/auxiliary_files. The original gene identifiers were updated to be consistent across datasets using the conversion table in auxiliary file allGenesOldIds.txt. If this updating merged multiple genes, the original expressions were averaged. Supplementary files are available under https://github.com/glaParaBio/deformable-infectious-pfalciparum-gams/tree/main/ref_data/extended_data.

## Footnotes

^1^
WHO. World malaria report 2025. 2025.
