# Supplementary material for: Defining the signature of deformable and infectious Plasmodium falciparum gametocytes
Source: Wellcome Open Res. 2026 May 14;11:156. Originally published 2026 Mar 2. [Version 3] doi: 10.12688/wellcomeopenres.25935.3 (PMC13126010; doi:10.12688/wellcomeopenres.25935.3)
Supplement: Peer review history

# Reviewer report: Reviewer response for version 3

**DOI:** 10.21956/wellcomeopenres.29357.r153940

**Published:** 2026-05-23

**Authors:** Nkechukwu Ikegbunam Moses

**Affiliations:** 1 Pharmaceutical Microbiology and Biotechnology, Nnamdi Azikiwe University Faculty of Pharmaceutical Sciences (Ringgold ID: 294839), Awka, Anambra, Nigeria

This is a clearly written and technically sound article addressing an important question in malaria transmission biology. The authors appropriately combine gametocyte deformability assays, exflagellation assays, mosquito feeding assays, and transcriptomic analysis to assess whether deformable Plasmodium falciparum gametocytes are already infectious.

 The study design is appropriate, the methods are sufficiently detailed, and the statistical analyses are suitable for the objectives of the work. The availability of source data and analysis code also supports reproducibility. The conclusions are adequately supported by the results.

 A few minor improvements could strengthen the article further.

 -The authors should maintain cautious wording around the claim of “immediate infectiousness,” since deformability and infectivity assays were not performed on the exact same sample set. 

 -They could also clarify the effect of dichotomizing continuous traits, such as exflagellation and oocyst density, and emphasize that the newly identified markers are promising candidates that still require wider validation.

 Overall, I recommend approval for indexing

Is the work clearly and accurately presented and does it cite the current literature?

Yes

If applicable, is the statistical analysis and its interpretation appropriate?

Yes

Are all the source data underlying the results available to ensure full reproducibility?

Yes

Is the study design appropriate and is the work technically sound?

Yes

Are the conclusions drawn adequately supported by the results?

Yes

Are sufficient details of methods and analysis provided to allow replication by others?

Yes

Reviewer Expertise:

Malaria molecular parasitology; Plasmodium falciparum molecular epidemiology; molecular diagnostics; antimalarial drug-resistance surveillance.

I confirm that I have read this submission and believe that I have an appropriate level of expertise to confirm that it is of an acceptable scientific standard.

# Reviewer report: Reviewer response for version 2

**DOI:** 10.21956/wellcomeopenres.29113.r153637

**Published:** 2026-04-29

**Authors:** Mariëtte van der Watt

**Affiliations:** 1 University of Pretoria, Pretoria, Gauteng, South Africa

I feel that this versions' aims and conclusions, although more concise, are far clearer. I have a few minor comments: 

 1) In the first "Results" paragraph, there is a statement: "Furthermore, at each time point, male gamete exflagellation was analysed (from

 upstream samples only, as there was insufficient yield from the downstream samples) and RNA was isolated". The last part makes it seem like RNA was isolated after the induction of exflagellation? 

 2) Although the DGE abbreviation is fixed (:-), it is not written out the first time it is used - see p6. 

 3) The last 2 sentences on p8, as well as the last sentence in paragraph 3 of p11 clarify a lot regarding the impact of the bin3 find. Thank you for adding these statements. My apologies if I missed them before. 

 4) Please remove the period from 10.000 rpm - p 13. 

 5) Top of p 14 - Please use a subscript for the "2"  in ddH2O and use either 10 ul or ten ul. This could be rewritten as "Sample (10 ul) was placed...".

Is the work clearly and accurately presented and does it cite the current literature?

Yes

If applicable, is the statistical analysis and its interpretation appropriate?

I cannot comment. A qualified statistician is required.

Are all the source data underlying the results available to ensure full reproducibility?

Yes

Is the study design appropriate and is the work technically sound?

Partly

Are the conclusions drawn adequately supported by the results?

Yes

Are sufficient details of methods and analysis provided to allow replication by others?

Partly

Reviewer Expertise:

Antimalarial drug discovery - was transmission focused earlier in my career.

I confirm that I have read this submission and believe that I have an appropriate level of expertise to confirm that it is of an acceptable scientific standard.

# Reviewer report: Reviewer response for version 2

**DOI:** 10.21956/wellcomeopenres.29113.r153636

**Published:** 2026-04-29

**Authors:** Antoine Dara

**Affiliations:** 1 PDNA, Pathogens genomic Diversity Network Africa (PDNA), Bamako, Mali, Mali

The authors addressed all my comments and concerns. No additional comments.

Is the work clearly and accurately presented and does it cite the current literature?

Yes

If applicable, is the statistical analysis and its interpretation appropriate?

Yes

Are all the source data underlying the results available to ensure full reproducibility?

Yes

Is the study design appropriate and is the work technically sound?

Yes

Are the conclusions drawn adequately supported by the results?

Yes

Are sufficient details of methods and analysis provided to allow replication by others?

Partly

Reviewer Expertise:

Molecular biology, bioinformatics, genomics, transcriptomics analyses (bulk and single cell RNAseq).

I confirm that I have read this submission and believe that I have an appropriate level of expertise to confirm that it is of an acceptable scientific standard.

# Reviewer report: Reviewer response for version 2

**DOI:** 10.21956/wellcomeopenres.29113.r153638

**Published:** 2026-04-28

**Authors:** Ibrahim bin Sa'id

**Affiliations:** 1 UIN Kediri, Kediri, Indonesia

I have now assessed the revised version. Overall, the manuscript has improved, and I appreciate the authors’ efforts in addressing the reviewers’ comments. In particular, I note improvements regarding the inclusion of more recent references and the clarification of the stages of gametocyte development, which were among my main concerns.

 Although I did not focus on detailed corrections per section or figure, my primary points of critique have been sufficiently addressed in this revision. Therefore, I am satisfied with the current version and would like to update my previous status from “Approved with reservations” to “Approved.”

 I would also like to highlight that this study is important, not only for advancing our understanding of Plasmodium falciparum, the most lethal malaria parasite, but also for its broader relevance to zoonotic malaria, which has been increasingly reported in several regions.

Is the work clearly and accurately presented and does it cite the current literature?

Partly

If applicable, is the statistical analysis and its interpretation appropriate?

I cannot comment. A qualified statistician is required.

Are all the source data underlying the results available to ensure full reproducibility?

Yes

Is the study design appropriate and is the work technically sound?

Partly

Are the conclusions drawn adequately supported by the results?

Partly

Are sufficient details of methods and analysis provided to allow replication by others?

Partly

Reviewer Expertise:

NA

I confirm that I have read this submission and believe that I have an appropriate level of expertise to confirm that it is of an acceptable scientific standard.

# Reviewer report: Reviewer response for version 1

**DOI:** 10.21956/wellcomeopenres.28546.r149777

**Published:** 2026-03-28

**Authors:** Antoine Dara

**Affiliations:** 1 PDNA, Pathogens genomic Diversity Network Africa (PDNA), Bamako, Mali, Mali

**Reviewer comments**

 The authors used a series of experiments including phenotypic traits and transcriptional profiling to describe *Plasmodium falciparum* gametocyte deformability and transcriptional signatures associated with infectivity. The work presented here has a potential to contribute malaria control and elimination efforts. However, there are some issues with the manuscript that should be addressed prior to indexing. Please find below some comments and suggestions for each section of the manuscript.

**Abstract**.

- The second sentence contains “progress” twice.

**Introduction section**

- Update the WHO reference with the latest 2025 report.
- In the 3 ^rd^ paragraph, it will be more useful to elaborate the gametocyte maturation process than the methods of detection and density threshold on the infectivity. What is the point of this paragraph is not clear to me? Maybe telling the reader that gametocyte density is important but does not tell of the full story about transmission and that other factors such morphological and transcriptional factors are required from successful transmission may help.
- First sentence of the last paragraph: suggest “… in blood circulation…” instead of “circulation” to be more specific.
- Be consistent in the spelling of “blood stream” with space and “bloodstream” without space.

**Results section**

- The description of all the figures should be complete and more self-explanatory, more comprehensive.
- Figure 1. The description of the figure is not complete. For instance, the time-course plot for gametocyte development in the panel A is not mentioned or explained in the text below the figure. The title should be “experimental setup” instead of “experimental plan”?
- Figure 2. The y-axes for the panels A and B are not indicated. In addition, the title of the figure does not capture the content. Please add the dynamics/trend aspects shown in the panel B.
- Figure 5. The description of the figure must be reviewed. For example, “ *Blue arrow in A and B shows patient samples with high cluster 95, 130, 191*”. There is no blue arrow in the figure 5.
- The sentence looks more like discussion. *“The reason for this difference in the number of DGE hits across infection traits may be biological but it could also have technical reasons, as parasite populations lose synchronicity with increasing duration of the experiment “.*
- Revise the following sentence *“Specifically, two gametocyte-specific clusters (95 and 130) and five clusters shared with the asexual stage (51, 97, 148, 161, 162, 191*)” in transcriptional network analysis subsection. The are 6 shared clusters that are listed instead of five.
- Also, use a uniform style for gene names “ *pfs25*, *pfs28, Pfs230p, Ccp4*, *pfmget* and *pfg377”. *Sometimes prefix “Pf vs pf”, *“ccp4 vs Ccp4*” etc.
- It is not clear, how the selection of the 3 putative markers for infectivity has been made. Why all the 3 markers have a negative DGE for deformability?
- The Spearman correlation between *bin3* expression and the proportion of infected mosquitoes is negative. Is that correct? If yes, this should be stated in the text to give the direction of the correlation.
- Please add a figure or supplemental showing a the pairwise comparison (correlation) between *ccp4*/ *mget* and *bin3 *expression.
- I also suggest to add a t-SNE plot to visualize the smart-seq2 single cell data to see if you can identity deformability or infectivity specific clusters (as supplemental figure).

**Method section**

- The format of the sub section 2 and 3 (…” *in vitro culture*” and “gametocyte culture”) should be the same as the others (same font and size).
- The sentence “For quantifying deformability and transcriptional profiling gametocytes were induced as previously described” should read “For quantifying deformability and transcriptional profiling, gametocytes were induced as previously described” with the coma after “profiling”. Similar sentences are found throughout the document.
- Microsphere filtration: “ Parasitemia of upstream and downstream samples was calculated, and the retention rate was calculated as 100 * (1- (downstream parasitemia/upstream parasitemia).” –missing parenthesis
- RNA-SPRI beads, SyberGreen, as well as the beads for microsphere filtration if available: indicate/provide the reference or catalogue number.
- RNA preparation: “ *by RNA 6000 Pico Bioanalyzer*”. Please rephrase it correctly. The RNA 6000 Pico is a kit and the 2100 BioAnalyzer is an instrument.
- Sentence beginning with digits should be spelled out. For example. “ *(…Ambion, AM9916). 10 μl of each sample …” *should reads “(…Ambion, AM9916). Ten μl (10µl) of each sample…”.
- RNA sequencing section. The last paragraph looks more like a result than the method. This should be revised. In addition, only the bioinformatics analyses should be described here.
- Phenotype and gene expression analysis:
  - “ *Describe where the count data and microarray data come from” *this looks like a comment
  - For the gene expression data, it is not clear how all the microarray datasets were normalized.
- Validating novel gametocyte markers: “From these samples, 11.5% (21/182) was infectious to mosquitoes.” should reads “From these samples, 11.5% (21/182) **were** infectious to mosquitoes.”

Overall, there are sentences with missing commas after introductory clauses to be corrected throughout the document. For instance, “ *To quantify the association between individual traits we used Spearman’s correlations* ( Figure 2C,D) in the second paragraph of the result section.” In addition, there are some elements of the discussion that are in the result section.

**Discussion**

 The discussion section needs to be more elaborated taking into account the other candidate markers. It is too focused on the bin3 that has been validated in a qPCR assay.

Is the work clearly and accurately presented and does it cite the current literature?

Yes

If applicable, is the statistical analysis and its interpretation appropriate?

Yes

Are all the source data underlying the results available to ensure full reproducibility?

Yes

Is the study design appropriate and is the work technically sound?

Yes

Are the conclusions drawn adequately supported by the results?

Yes

Are sufficient details of methods and analysis provided to allow replication by others?

Partly

Reviewer Expertise:

Molecular biology, bioinformatics, transcriptomics analyses (bulk and single cell RNAseq).

I confirm that I have read this submission and believe that I have an appropriate level of expertise to confirm that it is of an acceptable scientific standard, however I have significant reservations, as outlined above.

# Response

**Published:** 2026-04-04

**Authors:** Matthias Marti

**Affiliations:** University of Glasgow, UK

**Abstract**.

- The second sentence contains “progress” twice.

**Response:** This has been corrected. **Introduction section**

- Update the WHO reference with the latest 2025 report.

**Response: **The latest WHO reference has been added.

- In the 3 ^rd^ paragraph, it will be more useful to elaborate the gametocyte maturation process than the methods of detection and density threshold on the infectivity. What is the point of this paragraph is not clear to me? Maybe telling the reader that gametocyte density is important but does not tell of the full story about transmission and that other factors such morphological and transcriptional factors are required from successful transmission may help.

**Response:** We agree that the purpose of this paragraph was a bit unclear. We have added the following phrases to clarify: *“Hence, there may be additional factors apart from gametocyte density in the peripheral blood circulation that deter mosquito infectivity.”*

- First sentence of the last paragraph: suggest “… in blood circulation…” instead of “circulation” to be more specific.

Response: This has been corrected.

- Be consistent in the spelling of “blood stream” with space and “bloodstream” without space.

**Response:** This has been fixed.

**Results section**

- The description of all the figures should be complete and more self-explanatory, more comprehensive.
- Figure 1. The description of the figure is not complete. For instance, the time-course plot for gametocyte development in the panel A is not mentioned or explained in the text below the figure. The title should be “experimental setup” instead of “experimental plan”?

**Response:** The figure legend has been updated accordingly.

- Figure 2. The y-axes for the panels A and B are not indicated. In addition, the title of the figure does not capture the content. Please add the dynamics/trend aspects shown in the panel B.

**Response:** The figure legend has been updated accordingly.

- Figure 5. The description of the figure must be reviewed. For example, “ *Blue arrow in A and B shows patient samples with high cluster 95, 130, 191*”. There is no blue arrow in the figure 5.

**Response: **The figure legend has been updated accordingly.

- Revise the following sentence *“Specifically, two gametocyte-specific clusters (95 and 130) and five clusters shared with the asexual stage (51, 97, 148, 161, 162, 191*)” in transcriptional network analysis subsection. The are 6 shared clusters that are listed instead of five.

**Response:** This has been corrected.

- Also, use a uniform style for gene names “ *pfs25*, *pfs28, Pfs230p, Ccp4*, *pfmget* and *pfg377”. *Sometimes prefix “Pf vs pf”, *“ccp4 vs Ccp4*” etc.

**Response:** this has been corrected.

- It is not clear, how the selection of the 3 putative markers for infectivity has been made. Why all the 3 markers have a negative DGE for deformability?

**Response: **The rationale to select these three representative candidates is described in the previous results section: “Finally, we identified a shortlist of 92 candidate hits that were downregulated in deformable samples but showed a positive DGE association with either exflagellation or mosquito infection traits ( **Table S3**). If indeed infectious Stage V gametocytes represent a transcriptionally and physiologically different state from deformable Stage V gametocytes, then some of these candidate hits could represent this unique infectious state.”

- The Spearman correlation between *bin3* expression and the proportion of infected mosquitoes is negative. Is that correct? If yes, this should be stated in the text to give the direction of the correlation.

**Response:** This is correct. The correlation is negative for CT values and infectivity. We clarified that this indicated: “Higher CT values reflect lower transcript numbers; the correlation is thus in the same direction for bin3 as that for pfccp4/pfmget.”

- Please add a figure or supplemental showing a the pairwise comparison (correlation) between *ccp4*/ *mget* and *bin3 *expression.

**Response:** This has been provided. We agree that this direct correlation is of value. Including only assay positives, the correlation coefficient was -0.680 (p<0.0001). Among all samples (including negatives), the correlation coefficient was -0.303 (p=0.0037). The corresponding graph has been added as Figure 5D and a new legend is included. **Method section**

- The format of the sub section 2 and 3 (…” *in vitro culture*” and “gametocyte culture”) should be the same as the others (same font and size).

**Response:** This has been corrected.

- The sentence “For quantifying deformability and transcriptional profiling gametocytes were induced as previously described” should read “For quantifying deformability and transcriptional profiling, gametocytes were induced as previously described” with the coma after “profiling”. Similar sentences are found throughout the document.

**Response:** This has been corrected across multiple instances in the manuscript.

- Microsphere filtration: “ Parasitemia of upstream and downstream samples was calculated, and the retention rate was calculated as 100 * (1- (downstream parasitemia/upstream parasitemia).” –missing parenthesis

**Response:** This has been corrected.

- RNA-SPRI beads, SyberGreen, as well as the beads for microsphere filtration if available: indicate/provide the reference or catalogue number.

**Response:** This has been corrected.

- RNA preparation: “ *by RNA 6000 Pico Bioanalyzer*”. Please rephrase it correctly. The RNA 6000 Pico is a kit and the 2100 BioAnalyzer is an instrument.

**Response**: This has been corrected.

- Sentence beginning with digits should be spelled out. For example. “ *(…Ambion,* *AM9916* *). 10 μl of each sample …” *should reads “(…Ambion, AM9916). Ten μl (10µl) of each sample…”.

**Response: **This has been corrected.

- RNA sequencing section. The last paragraph looks more like a result than the method. This should be revised. In addition, only the bioinformatics analyses should be described here.

**Response:** We have left this section under methods as it doesn’t seem to fit under results.

- Phenotype and gene expression analysis:
  - “ *Describe where the count data and microarray data come from” *this looks like a comment

**Response: **The comment has been deleted.

- Validating novel gametocyte markers: “From these samples, 11.5% (21/182) was infectious to mosquitoes.” should reads “From these samples, 11.5% (21/182) **were** infectious to mosquitoes.”

**Response: **This has been corrected.

**Discussion**   The discussion section needs to be more elaborated taking into account the other candidate markers. It is too focused on the bin3 that has been validated in a qPCR assay.

**Response: **We added a statement that the current study does not provide evidence that *bin3 *is a better predictor of infectivity than other gametocyte marker. We consider an extensive discussion of molecular gametocyte markers to be beyond the scope of this manuscript. Moreover, such an overview has been provided elsewhere. *“Moreover, the current study did not include an extensive optimization of qPCR for other gametocyte markers. It is therefore possible that other markers, as identified in this study, are equally good or superior predictors of infectivity.”*

# Reviewer report: Reviewer response for version 1

**DOI:** 10.21956/wellcomeopenres.28546.r149779

**Published:** 2026-03-27

**Authors:** Ibrahim bin Sa'id

**Affiliations:** 1 UIN Kediri, Kediri, Indonesia

This work uses a combination of biomechanical assays, exflagellation measurements, mosquito feeding assays, and transcriptomic profiling to examine the link between deformability and infectivity of Plasmodium falciparum gametocytes. In addition to identifying transcriptional markers associated with transmission capability, the authors aim to determine whether deformable gametocytes correspond to infectious stages.

 The topic is important for understanding the biology of malaria transmission and may have implications for control strategies. A strength of the study is the integration of phenotypic and transcriptomic data. However, the main conclusions are undermined by several significant limitations in the study’s methodology, interpretation, and conceptual framing.

 The manuscript appears to rely heavily on earlier studies such as Lensen et al. (1999), which describe the dynamics of mosquito infectivity during in vitro gametocyte development, with a peak around days 10–12 of maturation. While this work is foundational, such reliance may lead to an oversimplified view of infectivity as a discrete or stage-dependent event. More recent studies, such as Joice et al. (2014), suggest that the acquisition of transmission competence is a gradual and progressive process, rather than an immediate transition.

 This is particularly relevant given that the authors frame their study around the late Stage IV to Stage V transition. However, the analysis of late-Stage IV gametocytes is not presented in detail, with most data appearing to reflect later time points dominated by Stage V. In addition, the use of 24-hour sampling intervals may be too coarse to capture rapid biological changes occurring during this transition, where finer temporal resolution (e.g., ~12-hour intervals) would be more appropriate.

 Taken together, these points suggest that the current interpretation may oversimplify a more complex biological trajectory, and that a more balanced integration of recent literature, together with a clearer treatment of Stage IV dynamics, would strengthen the manuscript.

 Finally, the use of the term “impossible” to describe the integration of deformability and infectivity measurements would benefit from clearer wording. This limitation seems to be due to practical and logistical challenges rather than a fundamental methodological issue. In principle, integration could still be considered, for example by using aliquots or parallel sampling from the same culture, although this may be technically challenging. In addition, the use of 24-hour intervals may not be sufficient to capture dynamic biological changes. Therefore, it would be better to replace “impossible” with a more appropriate term such as “technically challenging” or “beyond the scope of the study.”

Is the work clearly and accurately presented and does it cite the current literature?

Partly

If applicable, is the statistical analysis and its interpretation appropriate?

I cannot comment. A qualified statistician is required.

Are all the source data underlying the results available to ensure full reproducibility?

Yes

Is the study design appropriate and is the work technically sound?

Partly

Are the conclusions drawn adequately supported by the results?

Partly

Are sufficient details of methods and analysis provided to allow replication by others?

Partly

Reviewer Expertise:

malaria transmission biology, P. knowlesi, P. vivax, P. falciparum

I confirm that I have read this submission and believe that I have an appropriate level of expertise to confirm that it is of an acceptable scientific standard, however I have significant reservations, as outlined above.

## References

[1] : Plasmodium falciparum transmission stages accumulate in the human bone marrow. *Science Translational Medicine* .2014;6(244) : 10.1126/scitranslmed.3008882 10.1126/scitranslmed.3008882 PMC417539425009232

# Response

**Published:** 2026-04-04

**Authors:** Matthias Marti

**Affiliations:** University of Glasgow, UK

1. The manuscript appears to rely heavily on earlier studies such as Lensen et al. (1999), which describe the dynamics of mosquito infectivity during in vitro gametocyte development, with a peak around days 10–12 of maturation. While this work is foundational, such reliance may lead to an oversimplified view of infectivity as a discrete or stage-dependent event. More recent studies, such as Joice et al. (2014), suggest that the acquisition of transmission competence is a gradual and progressive process, rather than an immediate transition.

   This is particularly relevant given that the authors frame their study around the late Stage IV to Stage V transition. However, the analysis of late-Stage IV gametocytes is not presented in detail, with most data appearing to reflect later time points dominated by Stage V. In addition, the use of 24-hour sampling intervals may be too coarse to capture rapid biological changes occurring during this transition, where finer temporal resolution (e.g., ~12-hour intervals) would be more appropriate.

 Taken together, these points suggest that the current interpretation may oversimplify a more complex biological trajectory, and that a more balanced integration of recent literature, together with a clearer treatment of Stage IV dynamics, would strengthen the manuscript.

**RESPONSE:** This reviewer makes a fair point. Our data match the dynamics of infectivity during gametocyte development as observed by Lensen et al, which provides some cross validation for the 2 studies. However as discussed in the limitations section, our experiments and those presented by Lensen et al were performed under experimental conditions and therefore we cannot exclude requirement of additional parameters for development of infectious gametocytes during human infection. Of note, our previous work by Joice et al revealed tissue-specific enrichment of immature gametocytes in bone marrow but it did not provide any information on transmission competence. As for the Stage IV/V transition: it is defined by the deformability switch which we correlate for the first time with infectivity in the present study. Overall, our study adds a series of new parameters to correlate gametocyte stage development to mosquito infectivity. Based on these analyses we cannot support the

 2. Finally, the use of the term “impossible” to describe the integration of deformability and infectivity measurements would benefit from clearer wording. This limitation seems to be due to practical and logistical challenges rather than a fundamental methodological issue. In principle, integration could still be considered, for example by using aliquots or parallel sampling from the same culture, although this may be technically challenging. In addition, the use of 24-hour intervals may not be sufficient to capture dynamic biological changes. Therefore, it would be better to replace “impossible” with a more appropriate term such as “technically challenging” or “beyond the scope of the study.”

**Response:** This has been changed as suggested.

# Reviewer report: Reviewer response for version 1

**DOI:** 10.21956/wellcomeopenres.28546.r149771

**Published:** 2026-03-20

**Authors:** Mariëtte van der Watt

**Affiliations:** 1 University of Pretoria, Pretoria, Gauteng, South Africa

1. In the “Conclusions” section of the abstract, you use the term “unfiltered” to describe immature gametocytes. This is, apart from the methods section, the only place where you use this term. I feel like it confuses things. Perhaps rather stick to the term “stiff” or “rigid” used further along the paper.
2. In the plain language summary, replace “remained” with “remains”.

**Introduction**

1. The WHO reference can be replaced with the most recent one.
2. In the last sentence of paragraph one, I would replace “malaria” with “the disease” as I feel it would read better.

**Results/Discussion**

 Page 5, paragraph 3: is the acronym DGE correct?

 Page 10, second paragraph: It is not clear what you define as infectious vs. non-infectious patients. Is it based only on the presence of gametocytes in the blood samples? Also, why are the uncomplicated vs. cerebral nature of the two patient cohorts highlighted? Do you expect any differences in gametocyte carriage based on the severity or clinical symptoms of the disease?

 Page 11, paragraph 1: It is stated that “male traits such as DNA replication…and cilium genes were depleted” in deformable gametocytes. Is this not expected since these gametocytes have not yet been exposed to any of the factors initiating exflagellation (e.g. XA, drop in temperature, pH) yet?  

**Figures**

 Figure 1A: The gametocyte maturation timeline does not explain much around the induction process. Perhaps include e.g. the day of initiation of gametogenesis, day(s) NAG/heparin are added etc.

 Figure 3A: Spelling error on the bottom left label – should be “oocysts detected”.

 Figure 2/5: In these two figures r and rho are used interchangeably to denote correlation. I would would stick to one.

**Materials and Methods**

1. In “ *Plasmodium falciparum in vitro* culture”, strains should be singular since only strain NF54 is referenced.

The last part of the same section – “All parasites…containing 10% human serum” - should be moved to the section on gametocyte culture and exflagellation assays.

1. The fact that the two gametocyte induction processes used for gametocyte deformability/mosquito infectiousness/transcriptional profiling differ so vastly is concerning. The timing and process for initiation differs as well as the process of synchronising the cultures and preventing the reinvasion of asexual stage parasites (NAG vs. heparin). Would you not be concerned that these differences affect the viability/infectivity of the gametocytes?
2. “SybrGreen” should be replaced with “SYBR Green”.
3. Remove point from “10.000 rpm”.
4. The subscript below “Phenotype and gene expression analysis” should be removed.

In the same subsection, replace “grouped according **by** their phenotype” with “grouped according **to** their phenotype”.

1. “11.5% (21/182) **was **infectious” should be “11.5% (21/182) **were** infectious”

Is the work clearly and accurately presented and does it cite the current literature?

Yes

If applicable, is the statistical analysis and its interpretation appropriate?

I cannot comment. A qualified statistician is required.

Are all the source data underlying the results available to ensure full reproducibility?

Yes

Is the study design appropriate and is the work technically sound?

Partly

Are the conclusions drawn adequately supported by the results?

Yes

Are sufficient details of methods and analysis provided to allow replication by others?

Partly

Reviewer Expertise:

Antimalarial drug discovery - was transmission focused earlier in my career

I confirm that I have read this submission and believe that I have an appropriate level of expertise to confirm that it is of an acceptable scientific standard, however I have significant reservations, as outlined above.

# Response

**Published:** 2026-04-04

**Authors:** Matthias Marti

**Affiliations:** University of Glasgow, UK

**Abstract**

1. In the “Conclusions” section of the abstract, you use the term “unfiltered” to describe immature gametocytes. This is, apart from the methods section, the only place where you use this term. I feel like it confuses things. Perhaps rather stick to the term “stiff” or “rigid” used further along the paper.

**Response:** This has been changed as suggested. The phrase now reads: *“* *Our data revealed major transcriptional differences between input and deformable (i.e., filtered) gametocytes, but high similarity between deformable and infectious gametocytes.”* **Introduction**

1. The WHO reference can be replaced with the most recent one.

**Response:** This has been updated.

1. In the last sentence of paragraph one, I would replace “malaria” with “the disease” as I feel it would read better.

**Response: **This has been changed as suggested. **Results/Discussion**

 Page 5, paragraph 3: is the acronym DGE correct?

**Response:** DGE has been removed from this phrase.

 Page 10, second paragraph: It is not clear what you define as infectious vs. non-infectious patients. Is it based only on the presence of gametocytes in the blood samples? Also, why are the uncomplicated vs. cerebral nature of the two patient cohorts highlighted? Do you expect any differences in gametocyte carriage based on the severity or clinical symptoms of the disease?

**Response: **We have clarified this. There were two separate analyses. The first addressed whether putative markers of infectiousness are expressed during natural infections; the second directly determined the predictive value of one marker in a cohort of participants with known infectivity status. The uncomplicated and cerebral patients are used for the former analysis and a cohort of naturally infected Ugandans - many of whom were non-symptomatic – was used for the second analysis. During the revision, we clarified this by adding a new phrase and we also changed the heading of this paragraph to better reflect the three cohorts (i.e. ‘Analysis of putative infectivity markers in cohorts of naturally infected individuals’). New phrase: “ *Of note, these cohorts of patients from Malawi and Senegal had data on gametocyte carriage but not on infectivity to mosquitoes.”*  

 Page 11, paragraph 1: It is stated that “male traits such as DNA replication…and cilium genes were depleted” in deformable gametocytes. Is this not expected since these gametocytes have not yet been exposed to any of the factors initiating exflagellation (e.g. XA, drop in temperature, pH) yet?  

**Response:** this is a plausible explanation. We have added this to the revised manuscript that now reads “Notably, female gamete traits such as the crystalloid body were enriched in deformable gametocytes, while male traits such as DNA replication (occurring during gamete exflagellation) and cilium genes were depleted in these gametocytes that were not yet exposed to triggers for gamete formation”.

**Figures**

 Figure 1A: The gametocyte maturation timeline does not explain much around the induction process. Perhaps include e.g. the day of initiation of gametogenesis, day(s) NAG/heparin are added etc.

**Response:** This figure has been updated as suggested.

 Figure 3A: Spelling error on the bottom left label – should be “oocysts detected”.

**Response:** This has been corrected.

 Figure 2/5: In these two figures r and rho are used interchangeably to denote correlation. I would would stick to one.

**Response:** This has been corrected.

**Materials and Methods**

1. In “ *Plasmodium falciparum in vitro* culture”, strains should be singular since only strain NF54 is referenced.

**Response:** This has been corrected.

1. The last part of the same section – “All parasites…containing 10% human serum” - should be moved to the section on gametocyte culture and exflagellation assays.

           
**Response:** The section has been moved as suggested.

1. The fact that the two gametocyte induction processes used for gametocyte deformability/mosquito infectiousness/transcriptional profiling differ so vastly is concerning. The timing and process for initiation differs as well as the process of synchronising the cultures and preventing the reinvasion of asexual stage parasites (NAG vs. heparin). Would you not be concerned that these differences affect the viability/infectivity of the gametocytes?

**Response: **We acknowledge that differences in induction methods make it impossible to rule out a contribution of technical aspects to the observed transcriptional patterns. At the same time, both heparin and NAG are routinely used to remove asexual parasites with no reported effects on gametocytes, which is why they are both standard methods. The mosquito infections were set up as part of the routine infections to ensure they matched the standard oocyst densities (described in detail in DOI: 10.1186/s12936-021-03912-x). Furthermore, we have previously shown that the Albumax, used in deformability assays, gives similar infectivity when compared to aged-matched stress induced gametocytes (doi: 10.1016/j.molbiopara.2024.111634). Here, the only difference between methods is that the direct induction with Albumax on asexual parasites leads to higher total number of gametocytes due to higher commitment rates of the initial asexual culture, but the resulting mature gametocyte population does not receive Albumax and as such is not impacted by this media change. This may alleviate some concerns that NAG and heparin treatment have differential impacts on gametocytes. Our finding that transcription profiles in both set ups were not different strengthens the argument by showing no matter the set up the same results are obtained.

1. “SybrGreen” should be replaced with “SYBR Green”.

**Response: **This has been changed.

1. Remove point from “10.000 rpm”.

Response: This has been changed.

1. The subscript below “Phenotype and gene expression analysis” should be removed.

**Response:** This has been changed.  

1. In the same subsection, replace “grouped according by their phenotype” with “grouped according to their phenotype”.

           Response: This has been changed.

1. “11.5% (21/182) **was **infectious” should be “11.5% (21/182) **were**infectious”

**Response: **This has been changed.
